# Modeling trend changes in percent of under five-year-old children with malnutrition amongst 39 Asian countries from 1987 to 2016 via growth mixture model

**DOI:** 10.1186/s40795-022-00530-x

**Published:** 2022-04-29

**Authors:** Parisa Keshani, Hadi Raeisi Shahraki

**Affiliations:** 1 Shiraz HIV/AIDS Research Center, Institute of Health, Shiraz University of Medical Sciences, Shiraz, Iran; 2 Department of Epidemiology and Biostatistics, Faculty of Health, Shahrekord University of Medical Sciences, Rahmatieh Educational Complex, Shahrekord, Iran

**Keywords:** Asia, Children, Growth mixture model, Malnutrition, Trend

## Abstract

**Purpose:**

Malnutrition is an important public health issue and the main cause of child morbidity and mortality, especially in developing countries. The present study aimed to model trend changes in percentage of the malnourished children under 5 among 39 Asian countries during 1987 to 2016.

**Methods:**

Information about percentage of the malnourished under 5 years children based on under-weight (weight for age) malnutrition for 39 Asian countries were extracted from Gapminder web site during 1987 to 2016. To cluster Asian countries based on trend changes, Growth Mixture Model (GMM) was implemented. All the statistical analyses were performed in Mplus 7.4 software and *P* < 0.10 in likelihood ratio test (LRT) was considered as statistically significant.

**Results:**

Based on *P*-value of LRT, the model with 3 clusters was selected. Although, cluster 3 with 9 countries had higher intercept in 1987 and the worst situation in malnutrition, they gained a sharp decrease (− 0.93) in percentage of malnourished children under five annually. The slope of − 0.64 for cluster 1 countries indicate a moderate decrease annually in percent of children with malnutrition. The other 20 countries with slope of − 0.29 in their linear trend belonged to cluster 2 which shows slow decrease in the percentage of children with malnutrition.

**Conclusion:**

The investments in public health and education programs, as well as political commitment and government proper response in line with needs and demands are crucial to promote food security, nourishing diets and improving child nutrition. Certainly, most of them are still a long way from eradicating malnutrition.

## Introduction

Malnutrition is a critical public health challenge and the leading cause of child morbidity and mortality, especially in developing countries [1, 2]. Around 45% of deaths among children under 5 were due to under-nutrition, primarily affecting developing countries [3]. Underweight is a composite indicator, reflecting either past or present under-nutrition. It can result from both wasting and stunting [4]. Globally, 462 million are underweight. Its prevalence is 27.4% in children under 5 in South Asia [5]. More than 12 million children suffer from severe acute malnutrition in six Asian countries: Afghanistan, India, Bangladesh, Indonesia, Pakistan, and Yemen [6]. Often underweight is demonstrated by less visible micronutrient deficiencies, e.g., iron-deficiency anemia [4].

Regardless of where a child comes from, malnutrition is determined by different factors based on comprehensive strategies [1, 2, 7, 8]. Many countries have considered various programs and policies to reduce malnutrition. In 1983, UNICEF began dialogues to promote a child survival plan and a development revolution for children. GOBI-FFF was one of the leading UNICEF global policies for child health, consisting of growth monitoring, oral rehydration, breastfeeding, immunization, female education, family spacing, and food supplementation [9]. The scaling up Nutrition (SUN) movement was another primary driver of international commitment to reduce child malnutrition. More than 30 countries have joined it and implemented direct nutrition interventions [10–12]. According to the SUN movement, scaling up of evidence-based interventions, including integrating nutrition in efforts to improve gender equality, agriculture, food security, social protection, education, water and sanitation policy, health care, a substantial increase in domestic support, and external assistance for nutrition are quite urgent [12]. Food policy can increase the availability and affordability of healthy and nutritious foods and restrict advertising unhealthy foods. Poverty policy is also essential to increase the affordability of the poor population [13, 14]. In this way, some countries combat hunger, while others do not [1, 15]. Implemented strategies in different regions must be evaluated to mitigate malnutrition in Asia further. The present study aims to cluster trend changes in the percentage of the underweight (low weight for age) in children under five across 39 Asian countries from 1987 through 2016 to identify the main longitudinal patterns.

## Materials and methods

The data on the percentage of malnourished children under five based on weight for age for 39 Asian countries were extracted from the Gapminder website from 1987 through 2016. Prevalence of underweight children is the percentage of children under five whose weight for age is more than two standard deviations below the median for the international reference population aged 0–59 months. The data comes from the WHO’s new child growth standards released in 2006.

To cluster Asian countries based on trend changes in the percentage of children under five with malnutrition, the Growth Mixture Model (GMM) was implemented. This model estimates the number of unobserved longitudinal patterns (clusters) as a latent variable considering both the inter-individual and intra-individual variations over time. The GMM models estimate the response variable (in the current study percentage of the malnourished children under 5) in each cluster using the intercept and the slope of a linear trajectory [16].

In a GMM with k latent patterns, the following equations estimate intercept and slope in each pattern:

$${y}_{it}^k={\alpha}_{i0}^k+{\alpha}_{i1}^k{\lambda}_t^k+{\varepsilon}_{it}^k$$

$${\alpha}_{i0}^k={\alpha}_{00}^k+\sum_j{\beta}_{01j}^k{x}_j+{\varepsilon}_{i0}^k$$

$${\alpha}_{i1}^k={\alpha}_{10}^k+\sum_j{\beta}_{11j}^k{x}_j+{\varepsilon}_{i1}^k$$

Where α_00^k is the overall mean for the weight-for-age malnutrition in the kth pattern and α_10^k corresponds to the annual mean rate of changes in the weight for age malnutrition for the kth pattern.

Recently, GMM has been used to investigate different patterns of breast cancer incidence in Africa [17], to obtain the main patterns in the incidence of lung cancer in Europe [18], and to cluster the trend changes of the mean annual exposure to particulate matter with an aerodynamic diameter of fewer than 2.5 μm (PM2.5 particles) in the Middle East countries [19]. All the statistical analyses were performed in Mplus 7.4 software, and due to the small sample size, *P* < 0.10 in the likelihood ratio test (LRT) was considered statistically significant.

## Results

**Fig. 1:**
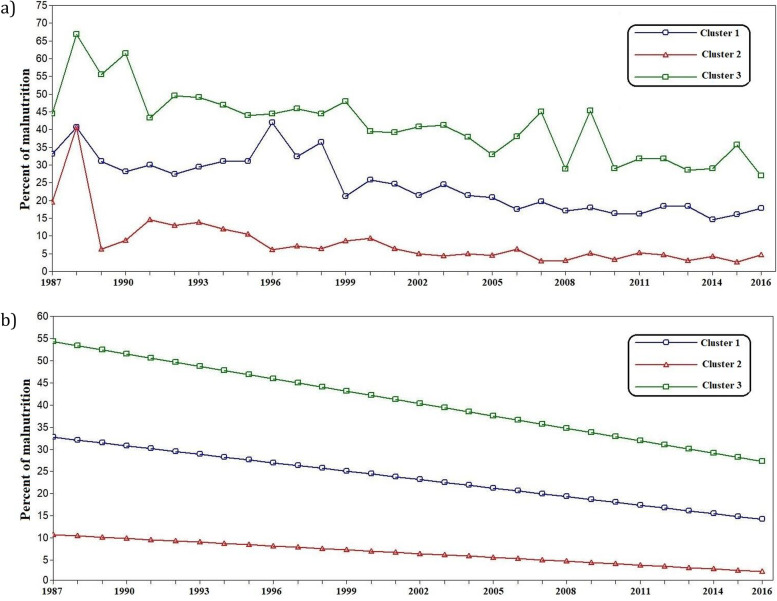
Overall mean (**a**) and estimated linear trend (**b**) of each cluster. Cluster 1 including: Bhutan, Indonesia, Malaysia, Maldives, Myanmar, North Korea, Philippines, Sri Lanka, Tajikistan and Vietnam. Cluster 2 including: Armenia, Azerbaijan, Bahrain, China, Iran, Iraq, Jordan, Kazakhstan, Kuwait, Kyrgyz Republic, Lebanon, Mongolia, Oman, Palestine, Saudi Arabia, South Korea, Syria, Thailand, Turkmenistan and Uzbekistan. Cluster 3 including: Afghanistan, Bangladesh, Cambodia, India, Lao, Nepal, Pakistan, Timor-Leste and Yemen

Table 1 reports the descriptive statistics of weight-for-age malnutrition among children under 5 for 39 Asian countries from 1987 to 2016 (in 5-year intervals). Although an overall decreasing trend has been evident for the past 40 years, all the Asian countries have not experienced the same trajectory. Thus, GMM was implemented to identify different unobserved patterns. GMM with different clusters were fitted, and the goodness of fit indices was calculated for all models. Finally, based on the *P*-value of LRT, the model with 3 clusters was selected (Table 2). The entropy of 0.995 indicated very high quality in the membership of clusters. As a result, the three primary patterns of sharp, moderate, and slow falling trends can summarize the observed longitudinal patterns in the percentage of the under-weight (weight for age) malnourished children under five in the Asian countries. Figure 1 displays the overall mean of each cluster and the estimated linear trends.

**Table 1:** Descriptive statistics of percent of malnutrition among under 5 year’s children for 39 Asian countries

Year	Min	Max	Mean	SD	Median
1987	18.7	66.8	35.3	15.2	33.4
1992	11.8	60.6	32.1	17.3	29.8
1997	3.8	52.5	32.8	17.6	37.6
2002	2.1	41.0	18.4	15.0	17.8
2007	2.2	48.6	21.7	16.4	20.4
2012	2.2	31.9	15.2	12.0	13.3
2016	2.6	27.0	14.9	9.1	16.3

**Table 2:** Results of the goodness of fit indices for different number of clusters

Fit indices	Number of classes							
	1	2	3	4	5	6	7	8
AIC	2210	1928	1735	1677	1610	1597	1579	1566
BIC	2263	1986	1799	1745	1683	1675	1662	1654
SSBIC	2163	1876	1680	1616	1546	1528	1505	1488
LRT P-value	–	0.39	0.05	0.12	0.20	0.39	0.67	0.40

Table 3 reports that the intercept and slope of linear trajectories also show the number of countries in each cluster. Although cluster 3 with nine countries, including Afghanistan, Bangladesh, Cambodia, India, Lao, Nepal, Pakistan, Timor-Leste, and Yemen, had higher intercept in 1987 and the worst situation in malnutrition, they gained a sharp decrease (− 0.93% per year) in the percentage of under-weight malnourished children under five annually. The slope of − 0.64 for cluster 1 countries, including Bhutan, Indonesia, Malaysia, Maldives, Myanmar, North Korea, Philippines, Sri Lanka, Tajikistan, and Vietnam, indicates a moderate annual decrease in the percent of children with weight for age malnutrition (0.64% per year).

**Table 3:** Intercept and slope of linear trajectories of growth mixture models for clustering of Asian countries based on percent of malnutrition

Cluster	Number of countries	Intercept		Slope	
		Estimate	SE	Estimate	SE
1	10	32.73	2.22	−0.64	0.11
2	20	10.76	2.90	−0.29	0.12
3	9	54.26	4.48	−0.93	0.17

The other 20 countries with a slope of − 0.29 in their linear trend belong to cluster 2, which shows a slight decrease in the percentage of children with malnutrition. Against the slow decreasing trend in cluster 2 (including Armenia, Azerbaijan, Bahrain, China, Iran, Iraq, Jordan, Kazakhstan, Kuwait, Kyrgyz Republic, Lebanon, Mongolia, Oman, Palestine, Saudi Arabia, South Korea, Syria, Thailand, Turkmenistan, and Uzbekistan) the current status of these countries is better than the countries in cluster 3 and 1.

## Discussion

Investigating underweight malnutrition among children under 5 years old in 39 Asian countries showed an overall decrease from 1987 to 2016. However, Asian countries had different reduction patterns due to experiencing some challenges, such as the economic crisis, which has aggravated malnutrition in 1997 and 2007, especially for clusters 1 and 3 [3]. It seems that one of the most critical sources of difference between the 3 clusters was the country’s economic crisis or good status in measures such as gross domestic product. We presented each cluster with examples from several countries in that group. The criteria for selecting countries in each category was access to more documentation and programs on child malnutrition. Furthermore, given that underweight is reflecting both acute and chronic malnutrition, reporting whole malnutrition-related results and strategies of the national interventions including stunting, wasting, underweight and micronutrient deficiencies was considered in our study.

Cluster 1 included Bhutan, Indonesia, Malaysia, Maldives, Myanmar, North Korea, Philippines, Sri Lanka, Tajikistan, and Vietnam, with a moderate decrease in the percent of underweight children.

Sri Lanka is a South Asian low-middle-income country with a high literacy rate [1]. Among children aged 6–59 months, underweight declined from 27.3 to 21% from 1996 to 2016. The improved nutrition was due to improved food availability, access and utilization, progress in health services, and water and sanitation and hygiene programs [20]. However, the micronutrient deficiencies among children remain persistent and intensified, especially by iron and vitamin A deficiencies [1]. The prevalence of anemia among children aged 6–59 months was 15.1%, 50% of it attributable to iron deficiency. Due to regional and gender disparities, the government should prioritize reducing income inequalities, assisting the most disadvantaged, creating productive employment, and reducing malnutrition, such as being underweight among children under 5 [20].

To end short-term hunger, increase school retention, and improve the food security and nutrition of school-age children, the strategic plan of Sri Lanka ensured receiving school meals for school-age children in high food insecurity and malnutrition areas all year round [20, 21]. Despite high literacy, economic growth, and successful programs, children’s nutritional status should still be improved [20, 21].

Like many other Asian countries, the Philippines suffers from micronutrient malnutrition. Implementing homestead food production (HFP) programs from 2003 to 2007 significantly decreased anemia prevalence among children (from 42.9 to 16.6%). HFP products also improved young children’s food security, nutrition status, and household income, significantly reducing the prevalence of anemia among children in the Philippines [22]. Moreover, political commitment measured by the Hunger and Nutrition Commitment Index (HANCI), was moderate [15].

The Philippines expanded governance through “Peace and Development Teams” (PDT) in 2002. The program progressively reduced child malnutrition by about 10% after the first year and 30% after 3 years, and its effects persist. PDT proxies for a general expansion of social services, security improvements, access to markets, or existing government services must be the causal mechanisms responsible for the malnutrition reduction [23].

The Philippines and Vietnam are en route to safe water and sanitation, elements recommended by the SUN movement; however, a country such as Indonesia has experienced slow progress in both domains, Myanmar has also acted slowly in providing safe water [14].

North Korea suffered from severe famine and food shortage during the 1990s, two leading causes being the yearly natural disasters, such as flood or drought, and the fall of the Soviet economic system. This shortage affected many areas, including child growth and mortality [24]. As children were most affected by food shortages and environmental changes, North Korea ranked first in child malnutrition [25]. Most of the interventions in North Korea have been conducted by UN agencies and NGOs; however, the scale of the interventions may not have been large enough to have had a meaningful impact. Moreover, access to the northern areas of North Korea, which remains the high malnutrition level in that region, is minimal. Despite all mentioned challenges, the nutritional status of children has recovered to a low and medium level for acute and chronic malnutrition status, from 1998 to 2012 that, both statuses can affect underweight [24].

Despite the sustained economic growth and progress in reducing poverty, child nutrition in Indonesia was in horrid conditions, with chronic malnutrition rates, which remain high. The recent 2013 Basic Health Survey (Riskesdas) in Indonesia found the prevalence of underweight among children under five to be 19.6%. The experience of Indonesia shows that economic growth alone is not enough to improve the nutritional status of children [26]. The massive expansion of village programs during 1975–90 covered all villages by 1990 with a steady decrease in underweight. Although the program ended, the planners now intended to restart it [7]. Most nutrition intervention programs only eliminate the symptoms without removing the causes of hunger. For policy planners and program designers tackling child malnutrition, it is now imperative to first understand the relationship between socio-economic characteristics and under-nutrition. Identifying socio-economic factors that significantly affect child nutrition status would provide valuable practical leads for combating the causes of child malnutrition in the country [26].

Twenty countries, including Armenia, Azerbaijan, Bahrain, China, Iran, Iraq, Jordan, Kazakhstan, Kuwait, Kyrgyz Republic, Lebanon, Mongolia, Oman, Palestine, Saudi Arabia, South Korea, Syria, Thailand, Turkmenistan, and Uzbekistan, were categorized in cluster two. Despite the slightest decrease, the current status of these countries is better than in other Asian countries.

Childhood malnutrition, including anemia and underweight, depicts a decreasing trend in China. Between 1990 and 2013, the rate of underweight children under five decreased from 13. 7 to 1.37% [27]. Such reduction was due to the governmental efforts to decrease poverty and an increased commitment to overall health improvements due to implementing programs such as China’s five-year plan for national development in the mid-2000s [28]. Over the past few years, due to the wide participation of the entire society and strong support of the international community, China has made notable progress not only in eliminating poverty and hunger but also in achieving universal primary education, ensuring healthcare for children, controlling and preventing diseases, and protecting the environment [27]. With nutrition improvement for children in poverty areas program in 2012, the National Health and Family Planning Commission (NHFPC) started promoting Ying Yang Bao, a soybean-based micronutrient-fortified food supplement with an effect on reducing anemia and other micronutrient deficiencies.

Moreover, the supplementation programs reduce significant micronutrient deficiencies in a short period, which localized approaches to maintain their gains. In addition, the advertising law in April 2015 was revised for infant formula, beverage, and other food, which claim its function as a replacement of breastfeeding [29]. Since 2011, the government has also planned a program to enhance nutrition for rural children receiving compulsory education, improve facilities in school dining halls, introduce nutritional meals, and gradually increase food allowances [27]. Nevertheless, anemia and malnutrition were prevalent among rural children compared to urban children. Overall, the difference in malnutrition between urban and rural children has also decreased with improved living conditions in the past 25 years [30, 31], but still, the micronutrient deficiencies are a child health problem [28].

The government of Kuwait also had its programs. It established a comprehensive social welfare program, including free housing and medical services for citizens, financial support of vulnerable groups, guaranteed employment, and free education until 15. Due to such programs and the discovery of oil resources, the country achieved socio-economic development, which considerably changed the food patterns and lifestyles of the people in Kuwait [32, 33]. As a result, data showed no under-nutrition and underweight among Kuwaiti preschool children [33, 34]. The experience of Kuwait highlights the importance of socio-economic development in overcoming malnutrition; however, strategies to decrease iron deficiency anemia are necessary.

In Iran, all types of malnutrition, including underweight, have improved among children under five due to the Iranian food and nutrition security system. Nationwide reports showed that the prevalence of child malnutrition had significantly reduced. The prevalence of underweight decreased from 13.8% in 1995 to 4.6% in 2004 [35]. Similarly, the malnutrition assessment revealed that all indicators of malnutrition had consistently decreased [36]. Like most Asian countries, malnutrition among rural children was more prevalent in Iran [35]. A multidisciplinary intervention to reduce protein-energy malnutrition among rural children was implemented from 1996 to 1999 through local nongovernmental organizations, including nutrition, health, and literacy education for mothers.

Moreover, improved growth monitoring and fostering rural cooperatives were considered [36]. Studies have shown that the primary policies related to Iranian child nutrition are divided into two policies consistent with the GOBI-FFF and policies focusing on the quality of life [9]. The most important policies and implemented programs were growth monitoring, oral rehydration, breastfeeding, female education, family spacing, food supplementation and enrichment, control of nutritional deficiencies, full coverage of free vaccination, eradication of primary contagious diseases, and free healthcare services for children through the health network system. Iran has also considered free screening of all preschool children [9, 37]. However, food security analysis shows a high variation of food security in Iranian provinces and weakness in tackling child micronutrient deficiencies, including iron, iodine, vitamin A, and vitamin D [9, 35]. There are adequate nutrition policies for food and nutrition security, even though there is no specific policy related to nutrition and prevention of malnutrition in children under five [9]. Therefore, despite the improving trend in child nutrition, the prevalence of child malnutrition is still three times more than the national average in deprived areas, and micronutrient deficiency is high among vulnerable groups [35, 38].

Although this category includes countries such as Syria and Iraq, they have struggled with the economic and political crisis due to war and conflicts that have affected health indicators. They could somewhat tackle malnutrition by supporting international organizations like UNICEF. In 1990, following the Gulf war and throughout the sanctions of the 1990s, UNICEF focused on rehabilitating social services, providing life-saving therapeutic feeding to malnourished and the most vulnerable children. In 1997, this organization again attempted to improve a child’s care via the “Oil for Food” program. As Syria’s conflict brought 250,000 refugees to Kurdistan, UNICEF established water and sanitation, education, health, and child protection services in 2012–2013. Since 2014, when internal conflict uprooted millions of Iraqis, UNICEF has implemented other programs to support children and families [39]. However, childhood malnutrition is still a significant public health problem in Baghdad. This experience showed that malnutrition is related to insecure living areas [40].

Cluster 3 includes Afghanistan, Bangladesh, Cambodia, India, Lao, Nepal, Pakistan, Timor-Leste, and Yemen, with the highest percent of malnutrition in 1987, which gained a steep decrease. Although these countries did very well in reducing undernourishment, malnutrition is still higher there than elsewhere. Therefore, they should perpetuate their effort and nutrition interventions.

The experience of the community-based program linked to nutrition in some of the Asian countries such as Bangladesh, India, and Thailand decreased malnutrition and increased children’s health and nutrition [41]. The homestead food production (HFP) program was successfully implemented in countries (e.g., Bangladesh, Nepal, and Cambodia) to increase the availability and intake of micronutrient-rich foods in poor households between 2003 and 2007. The program had a positive impact on the nutritional status of children and significantly improved dietary diversification. Moreover, the sale of HFP products improved household income and decreased anemia prevalence among children [22].

The Government of Bangladesh (GoB) has considered a National Food Policy since 2006 to enhance food availability, access, and utilization. In addition, Bangladesh joined the SUN movement in 2013 [10]. The expanded program on immunization, vitamin A supplementation, and high zinc treatment for diarrhea was also successful in Bangladesh. The GoB is scaling up its infant and young child feeding program throughout the country and provides community nutrition interventions through community clinics [42]. The exclusive breastfeeding for the first 6 months, followed by complementary feeding, was actively promoted, but GoB is still concerned about complying with the provisions of the international code of marketing of breast-milk substitutes in domestic law, are still concerns for [43]. Overall, although Bangladesh succeeded in reducing child malnutrition and declining poverty, it still faces hunger due to the high population growth and political instability, which affect public access to food [15]. This evidence shows the complexity of implementing interventions to reduce child malnutrition and reveals the effect of different factors on the interventions.

Political or government commitment was one of the critical elements to reduce malnutrition. According to Hunger and Nutrition Commitment Index (HANCI) most countries in this cluster, such as Pakistan, Yemen, and Afghanistan, had “low” to “very low” levels of political commitment [15].

As a middle-income country, India reported higher government commitment; however, this was not enough, and essential nutrition interventions did not cover half of the group for whom they were intended. Therefore, more committed action is required in India, focusing on malnourished children [15, 44]. Overall, the existing evidence showed that the prevalence of under-nutrition among under-five Indian children was high and varied widely. The diversity in covariates between the states clarifies the need for Indian nutrition programs to adopt state-specific approaches to tackle malnutrition. More focus on socio-economic development is required to overcome malnutrition [45].

The nutritional status of children in Nepal has also substantially improved over the past 15 years. Child underweight rates have decreased to about 29% of all children aged 6–59 months [46]. Success in reducing child undernutrition status was achieved by combining important health and nutrition strategies. Key factors were the community-based approach of programs, the improved coverage of safe motherhood programs, providing iron and folic acid supplementation to pregnant and breastfeeding mothers, and maternal care and child survival interventions. Despite breastfeeding in 70% of infants, only one-fourth of children aged 6–23 months were appropriately fed [15, 46].

The efforts of Yemen on hunger and nutrition were also deteriorating [15]. This condition might be due to the challenging period from 2011 until the start of the war in 2015 that Yemen has faced tremendous difficulties with economic and social consequences, and children were no exception to the impact of these shocks. To decrease the negative impact of the national crisis on households, the Social Welfare Fund (SWF) was the public cash transfer program in Yemen in 2011 [47]. However, the Yemen National Social Protection Monitoring Survey (NSPMS) found that 44% of the extremely poor were not covered by the SWF program [47].

Cambodia implemented a nutrition-sensitive agriculture project and nutrition education program conducted by the government, health volunteers, and local NGOs, targeting caregivers with a child between 5 and 18 months of age, with excellent potential to improve infant and young child feeding (IYCF) practices. Nutrition education intervention, especially maternal education, was also embedded in a food security project and was positively correlated with the child’s dietary diversity [47]. Cambodia increased levels of early initiation of breastfeeding, exclusive breastfeeding for the first 6 months, vitamin A supplementation, and iodized salt coverage to decrease under-nutrition [14].

Data was extracted from Gapminder website, as a rich and reliable data to identify systematic misconceptions about important global trends and proportions. The aggregation of the prevalence data is based on UNICEF, WHO, and the World Bank harmonized dataset (adjusted, comparable data) and methodology. However, data is not updated in some countries.

In conclusion, although economic growth is an underlying factor in tackling child malnutrition, it is not the only determinant, and other socio-cultural and political factors also play a part. The investments in public health and education programs, political commitment, and proper government response in line with needs and demands are crucial in promoting food security, nourishing diets, and improving child nutrition. To reduce under-nutrition commitment from governments, this should be the priority of political strategies, and health should be considered an inevitable element of each policy. Moreover, NGOs, industry, and society should take the challenges alongside other national and international organizations. The successful experiences and expanding community-based approaches in all Asian countries should not be neglected. In this way, rural areas should receive more attention than before due to the higher prevalence of malnutrition in such areas.

Overall, all Asian countries have attempted to reduce child malnutrition, mainly relying on global organizations’ policies. Indeed, most of them are still a long way from eradicating malnutrition. All the countries should emphasize the high micronutrient deficiencies to reduce them further.

## Data Availability

Data would be available on request from the corresponding author.

## References

[1] Akhtar S (2016). Malnutrition in South Asia—a critical reappraisal. Crit Rev Food Sci Nutr.

[2] Kavosi E, Rostami ZH, Kavosi Z, Nasihatkon A, Moghadami M, Heidari M (2014). Prevalence and determinants of under-nutrition among children under six: a cross-sectional survey in Fars province, Iran. Int J Health Policy Manag.

[3] Bhutta ZA, Bawany FA, Feroze A, Rizvi A (2008). Editors. The impact of the food and economic crisis on child health and nutrition. UNICEF conference.

[4] World Health Organization. Malnutrition. 2018. Retrived from https://www.who.int/news-room/fact-sheets/detail/malnutrition.

[5] The World Bank. Prevalence of underweight, weight for age (% of children under 5) - South Asia. 2022.

[6] Ahmed T, Hossain M, Mahfuz M, Choudhury N, Hossain MM, Bhandari N, et al. Severe acute malnutrition in Asia. Food Nutr Bull. 2014;35(2_suppl1):S14-S26. 10.1177/15648265140352S103 25069289

[7] Tasnim T. Determinants of Malnutrition in Children Under Five Years in Developing Countries: A Systematic Review. Indian J Public Health Res Dev. 2018;9(6):333–8.

[8] Bloem MW, de Pee S, Le Hop T, Khan NC, Laillou A, Minarto, et al. Key strategies to further reduce stunting in Southeast Asia: Lessons from the ASEAN countries workshop. Food Nutr Bull. 2013;34(2_suppl1):S8-S16. 10.1177/15648265130342S103 24049992

[9] Mohseni M, Aryankhesal A, Kalantari N (2019). Prevention of malnutrition among children under 5 years old in Iran: a policy analysis. PLoS One.

[10] SUN. SUN countries. Retrieved 10th March 2018, from http://scalingupnutrition.org/sun-countries. 2013.

[11] The LANCET. Maternal and Child Nutrition: Executive Summary of The Lancet Maternal and Child Nutrition Series, 2015. Retrived from: https://www.thelancet.com/pb/assets/raw/Lancet/stories/series/nutrition-eng.pdf.

[12] Nabarro D. Global child and maternal nutrition--the SUN rises. Comment Lancet. 2013;2:666–7. 10.1016/S0140-6736(13)61086-7 23746773

[13] te Lintelo DJ, Haddad LJ, Leavy J, Lakshman R (2014). Measuring the commitment to reduce hunger: a hunger reduction commitment index. Food Policy.

[14] Kolčić I. Double burden of malnutrition: A silent driver of double burden of disease in low–and middle–income countries. J Global Health. 2012;2(2). 10.7189/jogh.02.020303. 10.7189/jogh.02.020303 PMC3529312 23289074

[15] Dolf J.H. te Lintelo, Lawrence J. Haddad, Rajith Lakshman, Gatellier K. The Hunger And Nutrition Commitment Index (HANCI 2013): Measuring the political commitment to reduce hunger and undernutrition in developing countries. Evidence report no78 Retriverd from: https://reliefweb.int/sites/reliefweb.int/files/resources/ER78%20HANCI.pdf. 2014.

[16] Salari M, Kazemnejad A, Zayeri F (2017). Using growth mixture modeling for clustering Asian and north African countries on the road injury death trend (1990–2010). Oman Med J.

[17] Bahabin Boroujeni M, Mehrabani K, Raeisi SH. Clustering trend changes of lung Cancer incidence in Europe via the growth mixture model during 1990–2016. J Environ Public Health. 2021;2021. 10.1155/2021/8854446. 10.1155/2021/8854446 PMC8052171 33897783

[18] Taei N, Shahraki HR (2021). Identification of Main patterns in the incidence of gynecological cancers amongst the provinces in Iran. J Biostatistics Epidemiol.

[19] Mardani G, Faradonbeh MA, Kelishadrokhi ZF, Shahraki HR (2021). Modeling trend changes of mean annual exposure to PM 2.5 particles in the Middle East countries via growth mixture models. Arab J Geosci.

[20] World food programme. 2017. Sri Lanka Country Strategic Plan (2018–2022). Retrived from: https://documents.wfp.org/stellent/groups/public/documents/eb/wfp293168.pdf.

[21] Ministry of Healthcare and Nutrition oF Sri Lanka. (2010) National Nutrition Policy of Sri Lanka. Published in the extraordinary gazette No. 1639/5 of Democratic Socialist Republic of Sri Lanka dated 2010.02.02. retrived from: https://extranet.who.int/nutrition/gina/sites/default/files/LKA%202010%20Sri%20Lanka%20National%20Nutrition%20Policy-English_0.pdf.

[22] Talukder A, Haselow N, Osei A, Villate E, Reario D, Kroeun H, et al. Homestead food production model contributes to improved household food security and nutrition status of young children and women in poor populations. Lessons learned from scaling-up programs in Asia (Bangladesh, Cambodia, Nepal and Philippines). Field Actions Science Reports The Journal of Field Actions. 2010(Special Issue 1).

[23] Berman E, Downey M, Felter J (2016). Expanding governance as development: evidence on child nutrition in the Philippines.

[24] Lee S-K (2017). North Korean children: nutrition and growth. Ann Pediatric Endocrinol Metabol.

[25] United Nations Children’s Fund Child malnutrition. United Nations Children’s Fund. 2002 . Available from: https://www.unicef.org.

[26] De Silva I, Sumarto S (2018). Child malnutrition in Indonesia: can education, sanitation and healthcare augment the role of income?. J Int Dev.

[27] Ministry of Foreign Affairs of the People’s Republic of China, United Nations System in China. Report on China's Implementation of the Millennium Development Goals (2000–2015). 2015; 1–97. Retrived from: https://www.fmprc.gov.cn/mfa_eng/zxxx_662805/W020150730508595306242.pdf.

[28] Yue A, Marsh L, Zhou H, Medina A, Luo R, Shi Y (2016). Nutritional deficiencies, the absence of information and caregiver shortcomings: a qualitative analysis of infant feeding practices in rural China. PLoS One.

[29] Scherpbier RW (2016). China’s Progress and challenges in improving child nutrition. Biomed Environ Sci.

[30] Zhai L, Dong Y, Bai Y, Wei W, Jia L (2017). Trends in obesity, overweight, and malnutrition among children and adolescents in Shenyang, China in 2010 and 2014: a multiple cross-sectional study. BMC Public Health.

[31] Feng A, Wang L, Chen X, Liu X, Li L, Wang B (2015). Developmental origins of health and disease (DOHaD): implications for health and nutritional issues among rural children in China. Biosci Trends.

[32] Ministry of Health (MOH). 1996. Kuwait family health survey, 1996. Ministry of Health, State of Kuwait.

[33] FAO. Kuwait Nutrition Profile – Nutrition and Consumer Protection Division. Retrived from: http://www.fao.org/3/aq040e/aq040e.pdf. 2006.

[34] Kalendar SA (2011). Development and evaluation of Let's eat smart: a pilot school-based nutrition intervention for Elementry school children in Kuwait.

[35] Damari B, Abdollahi Z, Hajifaraji M, Rezazadeh A (2018). Nutrition and food security policy in the Islamic Republic of Iran: situation analysis and roadmap towards 2021. EMHJ-Eastern Mediterranean Health J.

[36] Sheikholeslam R, Kimiagar M, Siasi F, Abdollahi Z, Jazayeri A, Keyghobadi K (2004). Multidisciplinary intervention for reducing malnutrition among children in the Islamic Republic of Iran.

[37] Motlagh ME, Kelishadi R, Amirkhani MA, Ziaoddini H, Dashti M, Aminaee T (2011). Double burden of nutritional disorders in young Iranian children: findings of a nationwide screening survey. Public Health Nutr.

[38] National micronutrients survey of Iranians. Tehran: Nutrition improvement office: Ministry of Health and Medical Education; 2011.

[39] UNICEF. Role of UNICEF in Iraq. Retrived from: https://www.unicef.org/iraq/about-us. 2019.

[40] Ghazi HF, Mustafa J, Syed Aljunid ZM, Isa MAA. Malnutrition among 3 to 5 years old children in Baghdad city, Iraq: a cross-sectional study. J Health Popul Nutr. 2013;31(3):350. 10.3329/jhpn.v31i3.16827 PMC3805885 24288949

[41] Mason JB, Sanders D, Musgrove P, Galloway R. Community health and nutrition programs. Disease Control Priorities in Developing Countries 2nd edition: The International Bank for Reconstruction and Development/The World Bank; 2006.

[42] SUN. Compendium of Country Fiches, New York: Scaling Up Nutrition. Retrived from: http://scalingupnutrition.org/wp-content/uploads/2012/09/archived/compendiurm-of-countryfiches-ROME-VERSION.pdf 2011.

[43] Government of Bangladesh (2013). National food policy plan of action and country investment plan: monitoring report 2013.

[44] Purnima Menon, Neha Kohli, Mara Van Den Bold, Elisabeth Becker, Nicholas Nisbett, Lawrence James Haddad, et al. 25 years of scalng up, Nutrition and Health Interventions in Odisha, India retrived from: http://ebrary.ifpri.org/utils/getfile/collection/p15738coll2/id/130412/filename/130623.pdf. 2016.

[45] Sahu SK, Kumar SG, Bhat BV, Premarajan K, Sarkar S, Roy G, et al. Malnutrition among under-five children in India and strategies for control. J Natural Sci Biol Med. 2015;6(1):18. 10.4103/0976-9668.149072 PMC4367032 25810629

[46] UNICEF (2013). Improving child nutrition: the achievable imperative for global Progress.

[47] Reinbott A, Jordan I. Determinants of child malnutrition and infant and young child feeding approaches in Cambodia. Hidden Hunger. 115: Karger Publishers; 2016. p. 61–7. 10.1159/000444609 27197522

